# Enhanced Magnetic Properties of BiFeO_3_ Thin Films by Doping: Analysis of Structure and Morphology

**DOI:** 10.3390/nano8090711

**Published:** 2018-09-10

**Authors:** Yilin Zhang, Yuhan Wang, Ji Qi, Yu Tian, Mingjie Sun, Junkai Zhang, Tingjing Hu, Maobin Wei, Yanqing Liu, Jinghai Yang

**Affiliations:** 1 Key Laboratory of Functional Materials Physics and Chemistry of the Ministry of Education, Jilin Normal University, Changchun 130103, China; youyigexiaoxiong@126.com (Y.Z.); m17519912307@163.com (J.Q.); yh25330889@126.com (Y.T.); j56751136@163.com (M.S.); jk156895@126.com (J.Z.); tjhumars@126.com (T.H.); jlsdzccw@126.com (M.W.); 2 State Key Laboratory of Inorganic Synthesis and Preparative Chemistry, Jilin University, Changchun 130012, China; zjk8688@126.com

**Keywords:** BiFeO_3_, substitution, perovskite structure, ferromagnetic properties

## Abstract

The improvement of ferromagnetic properties is critical for the practical application of multiferroic materials, to be exact, BiFeO_3_ (BFO). Herein, we have investigated the evolution in the structure and morphology of Ho or/and Mn-doped thin films and the related diversification in ferromagnetic behavior. BFO, Bi_0.95_Ho_0.05_FeO_3_ (BHFO), BiFe_0.95_Mn_0.05_O_3_ (BFMO) and Bi_0.95_Ho_0.05_Fe_0.95_Mn_0.05_O_3_ (BHFMO) thin films are synthesized via the conventional sol-gel method. Density, size and phase structure are crucial to optimize the ferromagnetic properties. Specifically, under the applied magnetic field of 10 kOe, BHFO and BFMO thin films can produce obvious magnetic properties during magnetization and, additionally, doping with Ho and Mn (BHFMO) can achieve better magnetic properties. This enhancement is attributed to the lattice distortions caused by the ionic sizes difference between the doping agent and the host, the generation of the new exchange interactions and the inhibition of the antiferromagnetic spiral modulated spin structure. This study provides key insights of understanding the tunable ferromagnetic properties of co-doped BFO.

## 1. Introduction

Magnetoelectric multiferroic materials have attracted attention owing to the coexistence of ferroelectric and ferromagnetic ordering and exhibition of potential magnetoelectric coupling between them [1,2,3]. This not only provides an opportunity to probe fascinating physics but it can also be exploited for new categories of magnetoelectric equipment, such as high frequency filters, sensors or gas sensing applications [4,5]. BFO, as the only room temperature multiferroic material found so far and has attracted tremendous attention among the limited choices provided by multiferroic materials. It presents the coexistence of antiferromagnetism with Neel point (T_N_~643 K) and ferroelectricity with Curie point (T_C_~1103 K) at room temperature [6,7,8]. BFO is a rhombohedral ABO_3_-type perovskite with R3c space group [5,9]. The lattice parameters of the perovskite unit cell are a = b = 5.558 Å, c = 13.87 Å and the rhombohedral angle is 89.3–89.48° [2,10]. The ferroelectricity of BFO originates from the stereochemical hybridization of 6s^2^ lone-pair electrons with the 2p^6^ orbital of O^2−^ ion and the 6p^0^ orbital of Bi^3+^ ion [11]. The ferromagnetic properties are associated with the partially filled d orbital of the Fe^3+^ ion by Fe-O-Fe super-exchange interactions [12]. Owing to the presence of the typical impurity phase (Bi_2_O_3_/Fe_2_O_3_/Bi_25_FeO_40_) and inherent high leakage current of the reaction mechanism, so the preparation of pristine BFO is extremely difficult [13,14,15]. In addition to this, BFO possesses a G-type antiferromagnetic long-range space spiral modulated spin structure with a wavelength of 62 nm [16]. As a result, the existence of this spin structure impedes the observation of any linear magnetoelectric effect and net magnetization [17]. In addition to this, the problem of weak coupling between the polarization and magnetism properties of BFO systems remains a challenge that has to be overcome and makes it difficult to grope for basic antiferromagnetic and ferroelectric properties [18]. These issues inhibit the application of BFO for the manufacture of multifunctional devices. The introduction of suitable doped ion is generally deemed to be an effective way to modulate properties. Consequently, in order to improve the ferromagnetic and ferroelectric properties of BFO, many investigations have attempted A-site substitution by choosing trivalent rare-earth (Er^3+^, La^3+^, Sm^3+^, etc.) or divalent ions (Ba^2+^, Ca^2+^, Pb^2+^, etc.), B-site substitution by transition metal ions (Cr^3+^, Mn^4+^, Ti^4+^, etc.) and co-A-B-sites substitution (Eu-Mn, Ce-Zr, Sm-Zr, etc.) [7,15,16,17,19,20,21,22,23,24]. 

Among all kinds of doping studies at A-site, various reports on Ho^3+^ ions-doped BFO can excellently enhance electrical and magnetic properties, which result from the emersion of exchange interaction between 3d electrons of Fe^3+^ ions and 4f electrons of Ho^3+^ ions and the destruction of the spin cycloidal structure [9,25]. Rahimkhani et al. reported that the considerable enhancement magnetic properties of BFO are substituted by various contents of Ho, which may be attributed to structural phase transition by Ho substitution [26]. Song et al. observed that the remnant magnetization (Mr) of Bi_0.9_Ho_0.1_FeO_3_ sample is nearly 35 times as large as that of BFO ceramics by rapid liquid phase sintering [27]. And we noted that, the advantage of Mn doped BFO lies in that Mn possesses magnetic activity and the multivalent states of Mn ions enable the crystal to compensate for the charge [28]. Early studies on Mn^4+^ ions-doped BFO bulk materials showed Mn^4+^ ions substitution at Fe sites changed both the structural and electronic structures, which results in a phase transition and an increase in magnetization. So far, to the best of our knowledge, there are few reports on the magnetic behavior of Mn^2+^ ions-doped BFO thin film [13,19,26]. Chen et al. reported that the maximum in Mr (5.1 emu/cm^3^) could be observed for BFO thin film doped with Mn^2+^ ions of 0.10 concentration at 10 K [24]. Furthermore, Dutta et al. observed the magnetic properties of Zr^4+^ and Sm^3+^ co-doped BFO nanocrystalline (Mr = 0.409 emu/g) are superior to those of pure (Mr = 0.003 emu/g) and Zr^4+^/Sm^3+^ single (Mr = 0.116/0.067 emu/g) [15]. Mukherjee et al. reported the Y and Mn co-doping is a valid mean to redress various problems relevant to device application [16]. It can be noted that co-doping can improve the magnetization better than single doping.

Motivated by the above-mentioned issues, the magnetic properties of BHFO, BFMO and BHFMO thin films, synthesized by a simple sol-gel technique based on ethylene glycol, were investigated at room temperature and compared with pristine BFO thin film. Moreover, the effects of Ho or/and Mn on the surface morphology and crystal structure of BFO thin film are studied by using TEM/Raman/XRD and so forth. The purpose of this work is to comprehend the relationship between structural and magnetic properties of Ho^3+^ ions or/and Mn^2+^ ions-doped BFO thin film through thin film growth experiments in order to control magnetic properties of BFO. Remarkable improvement in the magnetic property is realized in the single and co-doped thin films compared to pristine BFO. The possible reasons for the enhancement of magnetic property have been investigated and discussed in detail.

## 2. Experimental Section

### 2.1. Materials Preparation

**Figure 1:**
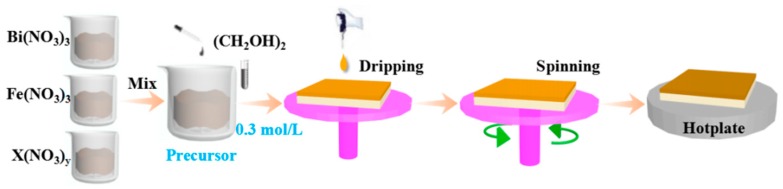
Schematic diagram of the process for preparing thin film via sol-gel technology.

Polycrystalline BFO, BHFO, BFMO and BHFMO thin films were synthesized on silicon substrates via a conventional sol-gel process. High purity grade Bi(NO_3_)_3_·5H_2_O, Fe(NO_3_)_3_·9H_2_O, Ho(NO_3_)_3_·5H_2_O as well as Mn(NO_3_)_2_·4H_2_O were used as starting materials. According to the composition formula BFO, BHFO, BFMO and BHFMO, all nitrates were dissolved in analytical grade ethylene glycol in proper proportion for the preparation of the precursor. The concentrations of bismuth and iron cations in the nitrate solutions were both 0.3 mol/L. The mixed solutions were stirred at room temperature for 6 h until the solution is clarified transparent and then aged in air for 24 h. The BFO, BHFO, BFMO and BHFMO thin films were deposited via spin coating the uniform precursor solution onto cleaned silicon (100) wafers at 1000 rpm for 3 s and 4000 rpm for 20 s and then baked at 350 °C on the hot plate. For each composition, the above processes were repeated for a couple of times until achieve the required thickness of the film. A schematic diagram of the above processes for preparing sample is shown in Figure 1. The dried deposited thin films annealed at 500 °C for 1 h at a heating rate of 2 °C min^−1^ in air and finally were cooled slowly to room temperature. 

### 2.2. Characterization

The structure and phase transformation characteristics of the BFO, BHFO, BFMO and BHFMO thin films were examined by X-ray diffraction (XRD, Rigaku Corporation, Tokyo, Japan) on a Rigaku D/MAX 3C X-ray diffractometer with Cu Kα radiation (λ = 1.5406 Å). The microstructure and component analysis of these samples were investigated by the Field Emission Scanning Electron Microscopy (SFEM, Model Hitachi S-570, JEOL Ltd., Tokyo, Japan) with Energy Dispersive Spectroscopy (EDS, JEOL Ltd., Tokyo, Japan) and a Transmission Electron Microscope (TEM, 200 keV, JEM-2100HR, JEOL Ltd., Tokyo, Japan). A PerkinElmer Nexion350-X inductively coupled plasma-mass spectrometer (ICP-MS, Agilent Technologies, PaloAlto, CA, USA) was used for elemental analysis. Raman spectra of all samples were measured in backscattering geometry using a Renishaw MicroRaman spectrometer with an excitation source of 514.5 nm (Renishaw, London, UK). The chemical composition and the valence state of all thin films were estimated through Escalab 250XI X-ray photoelectron spectrometer (XPS, Thermo Fisher Scientific, Waltham, MA, USA) using Mg Kα radiation (1253.6 eV) with a resolution of 1.0 eV. The magnetic properties of the BFO, BHFO, BFMO and BHFMO thin films were measured using a Lake Shore 7407 vibrating sample magnetometer (VSM, Lake Shore, Columbus, OH, USA) up to a maximum field of 10 kOe at room temperature. Temperature dependence of the magnetization was obtained using a magnetic property measurement system (MPMS, Quantum Design, SQUID-VSM, Inc., San Diego, CA, USA). 

## 3. Results and Discussion

**Figure 2:**
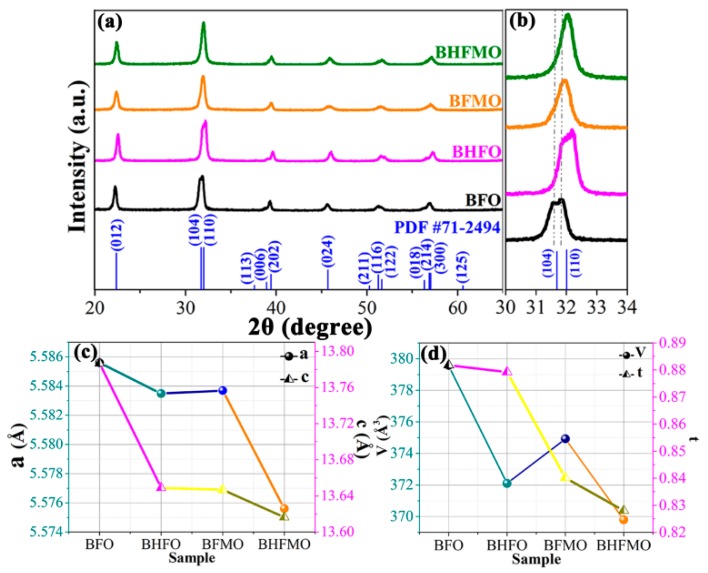
(**a**) X-ray diffraction (XRD) patterns of BiFeO_3_ (BFO), Bi_0.95_Ho_0.05_FeO_3_ (BHFO), BiFe_0.95_Mn_0.05_O_3_ (BFMO) and Bi_0.95_Ho_0.05_Fe_0.95_Mn_0.05_O_3_ (BHFMO) thin films; (**b**) The magnified patterns of the diffraction peaks located at around 32°; (**c**,**d**) calculated lattice parameters (**a**,**c**), cell volumes (V) and tolerance factors (t) as a function of all samples.

The XRD patterns of pure BFO as well as that of BHFO, BFMO and BHFMO thin films are recorded at room temperature are presented in Figure 2a. The diffraction peaks in the XRD pattern of BFO thin film are matched well with the standard diffraction pattern reported in JCPDS card No. 71-2494, which manifests the presence of a rhombohedral perovskite structure (R3c space group). The absence of peaks of the oxides of Ho or/and Mn predicates that substitution does not initiate the formation of these impurity phases. The amplified plot of the diffraction peaks located at 2θ ranges 31° to 34° is shown in Figure 2b to underline the evolution of the peaks (104) and (110) with Ho or/and Mn doping. These two peaks are clearly separated in BFO but show a trend towards coalescence in the BHFO and BFMO thin film. Eventually, the double-split peaks merge into a single peak in BHFMO thin film. These signs present a structural transformation between pure and substituted BFO thin film [2]. In addition to this, it is possible to expressly notice that all peaks have a significant shift to a high degree. The shifting of XRD peaks to the right in Figure 2b indicates the substitution of Mn and Ho into the Fe and Bi sites relative to the cubic perovskite parent structure, respectively. This phenomenon indicates that substitution induces lattice contraction, which can be attributed to the smaller sizes of holmium (R_Ho_^3+^, 1.015 Å) than that of the bismuth (R_Bi_^3+^, 1.08 Å) and the smaller sizes of manganese (R_Mn_^2+^, 0.67 Å and R_Mn_^3+^, 0.58 Å) than that of iron (R_Fe_^2+^, 0.76 Å and R_Fe_^3+^, 0.64 Å) ion [25,28]. See Figure 2c,d for changes in lattice parameters and cell volumes as the function of doping concentration. With Ho^3+^ or Mn^2+^ ions doping, the lattice parameter decreases as the unit cell volume decreases. Therefore, it is evident that Ho^3+^ or/and Mn^2+^ ions doping leads to lattice distortion in parent BFO thin film [9]. The decrease in the lattice parameters can be explained based on Goldschmidt tolerance factor (t), which is related to the radius of the cation between A and B ions.

(1) $$t=(R_{A}+R_{O})/2^{1/2}(R_{B}+R_{O})$$

The tolerance factors of all samples are presented in Figure 2d. The R_A_ and R_B_ are the radii of cations at A and B sites, respectively and R_O_ is the radii of anion at O sites. The smaller the tolerance factor, the bigger the FeO_6_ slope angle, also the more violent the distortion between the oxygen octahedra [29]. This is related to the smaller radii of ions at A/B-site cannot completely fill the empty space, which is manifested as an octahedron tilt, narrowing the space [18]. This induces lattice distortion inhibits the rhombohedral phase and causes the evolution of the lower symmetric phases like orthorhombic accompanied by the decrease in the unit cell volume. Therefore, these results manifest that the rhombohedral structure of BFO thin film will be changed by the substitution of Ho^3+^ or/and Mn^2+^ ions in the parent structure. The average crystallite size (D) is calculated by using the Debye-Scherrer’s formula:

(2) $$D=\frac{K\lambda }{\beta \cos\theta }$$

The average crystalline size is obtained to be 41 nm, 31 nm, 34 nm, 29 nm, respectively.

**Figure 3:**
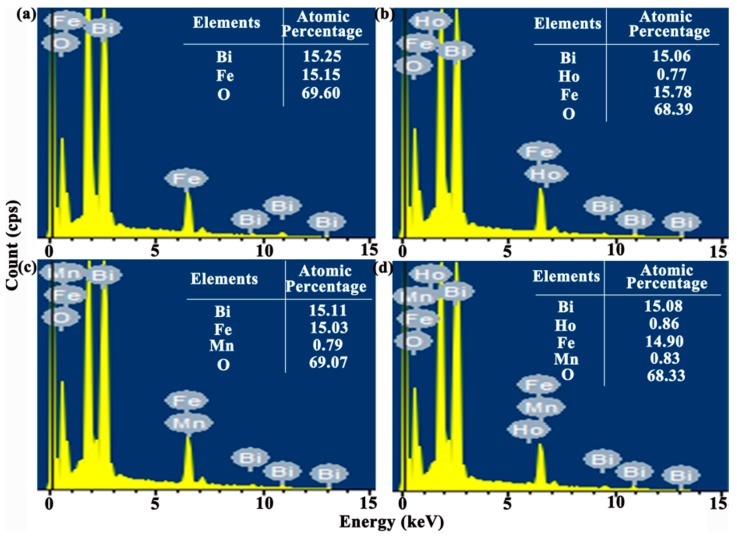
Energy dispersive spectroscopy (EDS) spectrum of (**a**) BFO (**b**) BHFO (**c**) BFMO (**d**) BHFMO thin film.

The energy dispersive spectroscopy (EDS) microanalysis (see Figure 3) connected to SEM is carried out with the working voltage of 15 kV to confirm the content of doping agent in the BFO thin film. Furthermore, the tables list the atomic percentages of all elements in the thin film. The experimental atomic percentage of Bi, Fe, O in pure BFO thin film is 15.25%, 15.15%, 69.60%. Under pristine BFO thin film circumstance, the atomic percent ratio of Bi/Fe is obtained approximately one. In addition, the actual concentration of Mn is around 0.0499 in the BFMO thin film and the actual concentration of Ho is around 0.0486 in the BHFO thin film. The actual concentrations of Ho and Mn are around 0.0540 and 0.0528 in the BHFMO thin film, respectively, which values approach the theoretical doping contents of Ho and Mn and confirming the success of Mn and Ho doping in the parent BFO thin film. In order to further confirm the element mass ratio, the content of the doping element in the thin films is quantitatively analyzed by ICP-MS. The sample is dissolved in concentrated HNO_3_ and then dilute to form solution at the concentration of 0.2 mg/L. The test results are summarized in Table 1. Based on the above results, it is clear that the chemical composition of all samples is close to the nominal composition, indicating that Ho or/and Mn ions are effectively incorporated into BFO thin film. 

**Figure 4:**
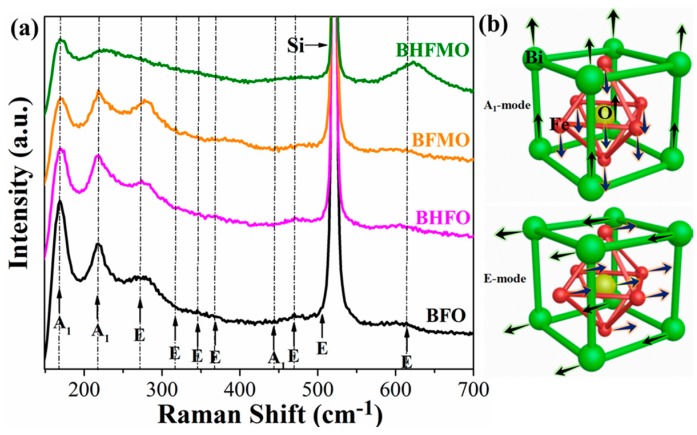
(**a**) Raman scattering diagrams of BFO, BHFO, BFMO and BHFMO thin films at the room temperature; (**b**) Illustrations show the A_1_ and E vibration normal modes for BFO.

Raman spectroscopy is used to further detect the structure and phase transition information of all thin films, as presented in Figure 4a. Group theory predicts that there are 13 active phonon modes (4A_1_ + 9E) with A_1_ and E symmetry in rhombohedrally distorted R3c space group for BFO [7,8]. Figure 4b presents the vibration modes of A_1_ and E. It can be observed that compared with the group theory, the BFO thin film in this paper shows less Raman active modes. This may be due to some Raman active modes of weakness are hard to observe. Raghavan et al. [29] revealed that A_1_ modes of the low-frequency region are responsible for the Bi–O vibrations and the E modes of the high-frequency region are associated with Fe–O vibrations. The observed low frequency modes move toward a higher frequency in the Raman spectra of BHFO thin film, which can be associated with the reduction of the stereochemical activity of the Ho-doped Bi 6s^2^ lone pairs [9]. Meanwhile, it can be found that A_1_ modes intensity decline along with increasing doping content of Ho^3+^ ions. A_1_ phonon modes are interrelated to Bi–O bonds [29]. Therefore, the decrease of A1 phonon modes means that the Ho-doped causes a significant change in Bi–O bonds. After doping Mn^2+^ ions, it can be found that the reduction of the A_1_ modes and enhancement of the E modes in BFMO thin film. The variety of E modes can be regarded as the bending and stretching of FeO_6_ octahedra [30]. In addition, the Raman scattering spectra in the vicinity of 624 cm^−1^ is changed significantly in E mode, which is related to the altered of Fe–O bonds induced by doping Mn^2+^ ions [30]. This result is in accordance with the reduced lattice parameter c in BFMO thin film of Figure 2d. In our samples, XRD data is supported due to the absence of Raman mode of the impurity phases.

**Figure 5:**
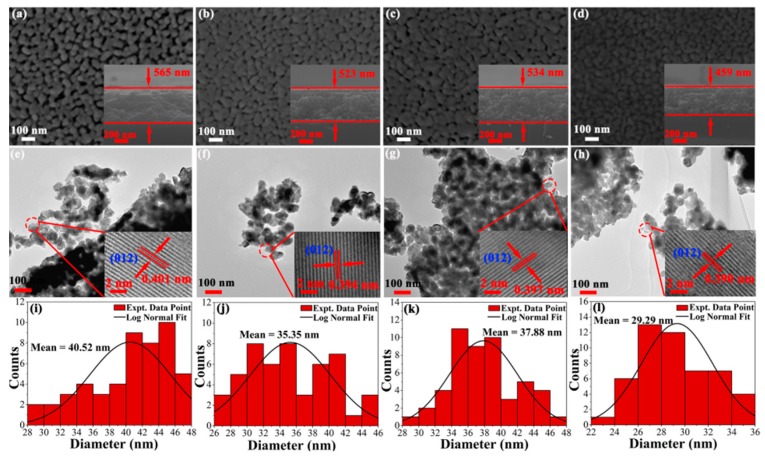
SEM images of surface morphologies for (**a**) BFO (**b**) BHFO (**c**) BFMO (**d**) BHFMO thin film and the insets in (**a**–**d**) show cross-sectionals of all thin films; (**e**–**h**) low magnification TEM images of BFO, BHFO, BFMO, BHFMO thin films and the insets in (**e**–**h**) show high resolution TEM images of all thin films; Histograms regarding particle size distributions of (**i**) BFO (**j**) BHFO (**k**) BFMO (**l**) BHFMO thin film, respectively.

Figure 5a–d shows the typical surface and cross-section micrographs of pristine and doped BFO thin film obtained by using a FE-SEM. The cross-sectional thickness is determined by FE-SEM micrographs with BFO thin film of 565 nm, BHFO thin film of 534 nm, BFMO thin film of 523 nm and BHFMO thin film of 459 nm. From the vertical view of typical surface morphology, wide porosity is observed between particles of BFO thin film. Notably, BHFO and BFMO thin films exhibit smaller porosity and denser morphology compare with pure BFO thin film. It is observed that the BHFMO thin film possesses a substantial reduction in pores and a compact packed in microstructure. This indicates that the Ho^3+^ and Mn^2+^ ions are extremely valid in restraint of particle growth [18]. The particle size distribution of the particles calculated for each sample is shown below in the corresponding SEM image (see Figure 5a–d. The average size of particles is 40.52 nm, 35.35 nm, 37.88 nm and 29.29 nm respective to pristine, BHFO, BFMO and BHFMO thin films. After doping with Ho^3+^ and Mn^2+^ ions, the particle size is found to be decreased, which is basically consistent with the calculated average crystalline sizes are given in Equation (2). Undoubtedly, the reduction of particle size in BHFO, BFMO and BHFMO thin films can be explained by the decreased crystal unit cell volume, similar to the observation in Ca-doped BFO [10]. The difference in doping content and ionic radius results in different degrees of distortion for BFO thin film and so the particle size is different. The doping content is the same in BHFO and BFMO thin film, so the factor that determines the difference in size is the ionic radius. However, Ho and Mn are co-doped in BHFMO thin film, so the factors determining the size of BHFO thin film are ion radius and total doping content. The size of Ho is smaller than that of the Bi and the size of Mn is smaller than that of Fe ion. Therefore, the particles vary in size for BHFO, BFMO and BHFMO thin films. In addition to this, both transition metal and rare earth ions can also serve as nucleation centers of perovskite structure and thereby increasing the nucleation rate and the number of nuclei immensely [13]. Figure 5e–h indicate the TEM micrographs of BFO, BHFO, BFMO and double-doped thin film the whole morphology in low-magnification image. The grain size seems to be decreased slightly with the substitution of the Ho^3+^ and Mn^2+^ ions. This is excellent consistent with the size acquired from SEM pattern recorded from the same thin film. The HRTEM images as shown in the inset are taken from the circular region as shown in Figure 5e–h. These images show that the spacing of the interplanar plane of a typical crystalline region is about 0.401 nm, 0.394 nm, 0.397 nm and 0.390 nm, which correspond to the (012) plane of BFO thin film (card #71-2494) [10].

**Figure 6:**
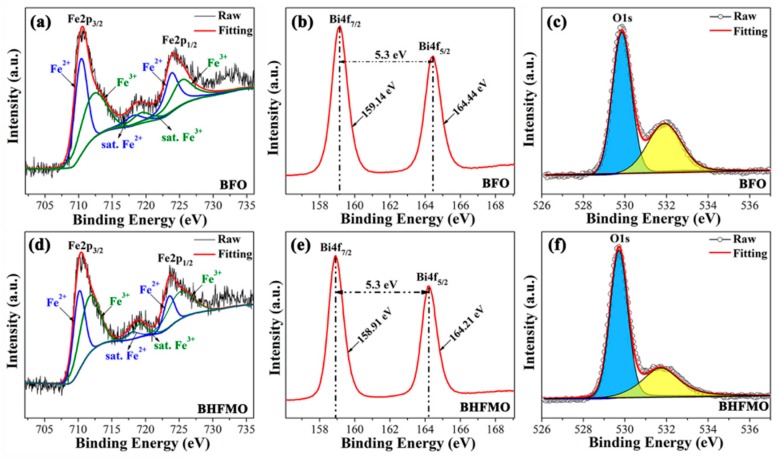
The X-ray photoelectron spectrometer (XPS) spectra of (**a**) Fe2p; (**b**) Bi4f and (**c**) O1s for the pure BFO thin film; XPS spectra of (**d**) Fe2p; (**e**) Bi4f and (**f**) O1s for the BHFMO thin film.

In the previous literature, it is known that the deviation from oxygen stoichiometry leads to valence fluctuation of Fe ions (+3 to +2 state) in BFO [31]. Pure BFO shows poor properties due to due to the oxygen vacancies derived from the reduction of Fe^3+^ to Fe^2+^ and Bi^3+^ volatilization [32]. For the sake of determining the elements, elemental oxidation states, chemical shifts and oxygen vacancies in doped BFO thin film, detailed XPS measurements are also carried out. Figure 6a-e shows the XPS spectra of the Fe, Bi and O regions for BFO and BHFMO thin films, respectively, where the core energy level C 1s (285.8 eV) peak is served as a reference for the correct of the binding energy values gained for the elements. In Figure 6a,d, the Fe 2p regions are fitted for BFO and double-doped thin films. The Fe2p splits into two peaks for 2p_1/2_ and 2p_3/2_ spin-orbit doublet components in pristine BFO and BHFMO thin films. Based on the Gaussian-Lorentzian curve fitting, it is found that the peaks at 712.57 eV, 719.46 eV and 725.67 eV belong to Fe^3+^ (green line), while the Fe^2+^ (blue line) locates at the peaks of 710.44 eV, 718.35 eV and 723.91 eV, which manifests the coexistence of Fe^3+^ and Fe^2+^ ions [17]. It is known that a slight oxygen deficiency in BFO can result in the formation of Fe^2+^ ions within the iron sublattice. Equations (3)–(6) indicate the creation of Fe^2+^ ions, as shown in Reference [33]:

(3) $$O_{O}\to V_{O}+\frac{1}{2}O_{2}↑$$

(4) $$V_{O}\to V_{O}^{•}+e^{\prime }$$

(5) $$V_{O}^{•}\to V_{O}^{••}+e^{\prime }$$

(6) $$Fe^{3+}+e^{\prime }\to Fe^{2+}$$

On the basis of the fitting, the specific value of Fe^3+^:Fe^2+^ is calculated to be 1.13 and 2.71 for BFO and double-doped thin films, respectively. The XPS spectrum of O 1s for BFO thin film is shown in Figure 6c, which can be fitted into two sub-peaks located at about 531.90 eV and 529.85 eV, respectively, attributing to the characteristic of O defects (yellow area) and lattice oxygens (blue area), respectively [17]. Notably, it is seen that the cation-oxygen bonds are around 85.1% in BHFMO thin film, which indicates the presence of a small amount of oxygen vacancies in BHFMO thin film [17]. From Figure 6b, we can see that Bi4f spectrum consists of two peaks at 159.01 eV for Bi4f_7/2_ and 164.31 eV for Bi4f_5/2_, which can be assigned to Bi-O bonds [21]. Figure 6e demonstrates that compared with the pure Bi4f spectrum, Bi4f_7/2_ (158.92 eV) and Bi4f_5/2_ (164.22 eV) peaks of the BHFMO thin film. The gap between the two peaks is approximately 5.3 eV, confirming the Bi^3+^ valence states in the BFO and BHFMO thin film [6]. Based on the above results, it is found that Ho and Mn co-doping can effectively restrain the formation of Fe^2+^ and the volatilization of Bi^3+^ in BFO, leading to the reduction of the oxygen vacancy. In the molecular formula of BFO, the valence state of Fe is trivalent and the magnetic properties of BFO from Fe. As described in the introduction, the ferromagnetic properties are associated to the partially filled d orbital of Fe^3+^ ion by Fe–O–Fe super-exchange interactions. The increase in Fe^3+^ ion will make the Fe–O–Fe super-exchange effect stronger. Fe^3+^ ions and Low oxygen vacancies play an active role in the BFO structure and affect the display of magnetic properties.

**Figure 7:**
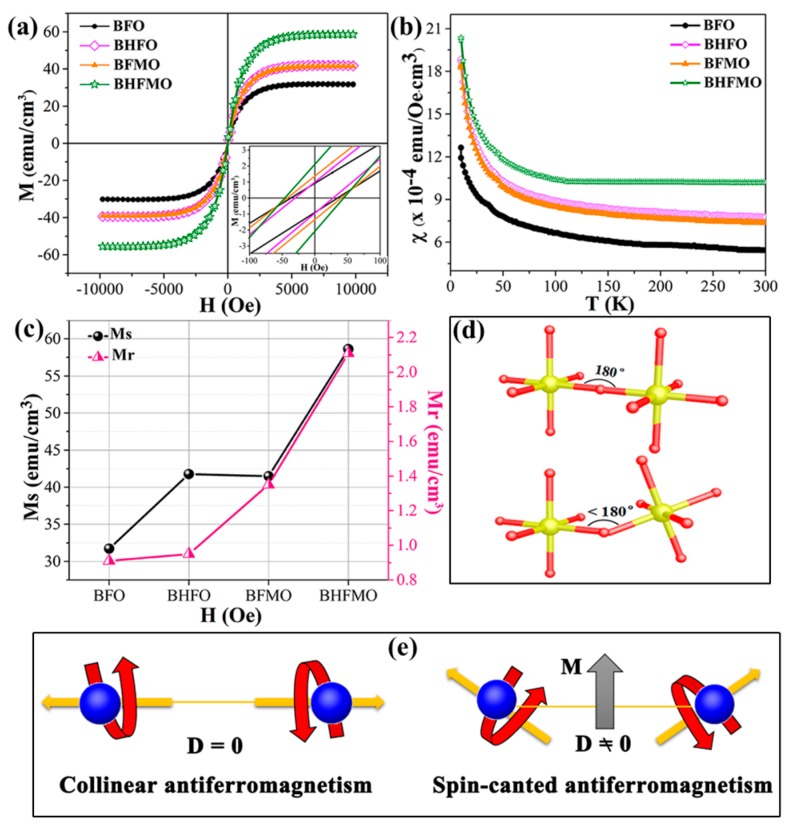
(**a**) Magnetic hysteresis loops measured at room temperature with the field applied in the plane for BFO, BHFO, BFMO and BHFMO thin films; The inset in (**a**) shows the enlarged M-H loop of all thin films; (**b**) Temperature dependence of magnetic susceptibility of BFO, BHFO, BFMO, BHFMO thin films as measured in an external magnetic field of 1000 Oe. For these measurements field-cooled (FC) conditions have been chosen; (**c**) The tendency of Mr and Ms for these samples; (**d**) Fe-O-Fe super-exchange interaction in the BFO and doped BFO thin films; (**e**) Canted antiferromagnetism spin configuration causes net magnetization in doped BFO thin film by the Dzyaloshinskii-Moriya interaction.

The structural measurements reveal the varieties in the aspects of magnetic properties for thin film. In order to quantify them, magnetic measurements at room temperature are tested. The magnetization hysteresis (M–H) loops of the BFO, BHFO, BFMO and BHFMO thin films at room temperature with an applied magnetic field of 10 kOe are presented in Figure 7a. The value of Ms is ascertained by the nodal increment of the two straight lines drawn by the high field and low field positions of M–H hysteresis loop. The calculated values of remnant magnetization (Mr) along with saturation magnetization (Ms) of all thin films are shown in Figure 7c. Obviously, pristine BFO thin film displays low spontaneous magnetization with a small Ms value 31.37 emu/cm^3^. This phenomenon may be due to the spin modulation spiral structure which hinders the observation of net magnetization [34]. The Ms of BFO with a doping concentration of 0.05 for Ho (41.78 emu/cm^3^) and Mn (41.48 emu/cm^3^) is basically the same as that for pure phase (31.70 emu/cm^3^). Compared with above those two dopants, the double-doped BFO thin film displays much stronger magnetization (58.62 emu/cm^3^). The inset in Figure 7a shows a zoomed-in view of the magnetic field representing the Mr. The illustration in Figure 7c shows the Mr of BFO, BHFO, BFMO, BHFMO thin films is 0.91, 0.95, 1.35 and 2.11 emu/cm^3^, respectively. There is a small increase in the Mr of Ho and Mn doping compared with Ms of single doped BFO thin film in bismuth ferrite system. Figure 7b shows the magnetic susceptibility of BFO, BHFO, BFMO, BHFMO thin films from 10 K to 300 K. For pure BFO thin film, the magnetization decreases slowly with decreasing temperature from 300 K to 40 K. As the temperature is further reduced, the magnetization increases obviously, which is the characteristic of weak ferromagnetism. Due to destruction of the spin cycloid structures, small magnetization exists in BHFO, BFMO and BHFMO thin films. The magnetization of these samples increases slowly with decreasing temperature between 40 K and 300 K. When the temperature is lower than 40 K, the magnetization increases sharply, indicating that the material BHFO, BFMO, BHFMO thin films have ferromagnetic properties. A careful inspection in the Figure 7b reveals that the magnetization increases with addition of Ho or/and Mn simultaneously at room temperature.

As is known to all, the macroscopic magnetization of BFO is eliminated because of the incommensurate cycloid spin modulation with a 62 nm wavelength [4]. It has been found that the magnetic properties will be favored by uncompensated spins when the typical size is reduced below 62 nm, due to the spiral spin structure can be partially destroyed [6,10]. The enhanced magnetic properties in BHFO thin film can be explained as: (i) owing to the change of spin structure of BFO, the long-range spiral modulation and the ferromagnetic properties of thin films are enhanced for the particles with sizes less than 62 nm [33,34]. The size of particles is within the region of 25–45 nm, which is smaller than that of the period length of 62 nm for the BFO. Hence, it will cause inferior ferromagnetic behavior, due to the long wavelength period of BFO thin film is partially wrecked, resulting in incomplete uncompensated spins being measurable [20,34]. (ii) ionic sizes of the holmium (R_Ho_^3+^, 1.015 Å) is smaller than that of the bismuth (R_Bi_^3+^, 1.08 Å) [18]. This results in changes in the Fe–O–Fe bond angles and Fe-O bond lengths (Figure 7d), as well as an increase in octahedral tilt, which in turn restrains the spatially modulated and releases the magnetism [35] In BFO, the first principle calculation reveals that the Fe–O–Fe bond angle is one of the critical parameters from the anti-ferromagnetic phase to ferromagnetic phase. The tilts of the Fe^3+^ ions spins are associated to the Dzyaloshinskii-Moriya (DM) interaction energy of Fe^3+^ ions (H_DM_):

(7) $$H_{DM}=\overset{n}{\underset{n=0}{\Sigma }}{\vec{D}}_{n}•({\vec{S}}_{0}\times {\vec{S}}_{n})$$

where ${\vec{D}}_{n}=V_{0}[\overset{}{{\vec{r}}_{n-0}}\times {\vec{r}}_{n-n}]$ is the interaction parameter of DM interaction, ${\vec{r}}_{n-0}$, ${\vec{r}}_{n-n}$ are the position vectors of the nearest neighbor magnetic ions from the nth O ions to the nearest magnetic Fe ions and *V*_0_ is the microscopic constant. ${\vec{S}}_{0}$ and ${\vec{S}}_{n}$ are the vectors of two Fe ions magnetic moments [36]. For the ideal state of the perovskite structure, since the Fe–O–Fe bond angle (φ) is equal to 180°, so the $\left[{\vec{r}}_{n-0}\times {\vec{r}}_{n-n}\right]$ factor is zero [37]. Thus, the anti-symmetric exchange energy term H_DM_ is zero. Nevertheless, if the φ begins to deflect from the ideality of 180°, the H_DM_ will increase, as shown in Figure 7e. Therefore, it is expected that the magnetization value will increase; and (iii) Ho^3+^ ions are active magnetic ions with large magnetic moments and ferromagnetic couplings of Ho^3+^ and Fe^3+^ ions may administer to increase the magnetization value [38]. 

The Mn substitution of the Fe position not only helps to suppress spiral spin structure but also facilitates the new magnetic exchange mechanism between Fe^3+^ and Mn^2+^ ions [13]. As the neighbor of the surrounding Fe ions, the Mn ions do not eliminate the magnetic spin of the Fe sublattice but can help improve the degree of spin canting of the surroundings in a way, thus enhancing the magnetization [24]. Above all, the coexistence of Fe^3+^ and Mn^2+^ ions causes a ferromagnetic Fe^3+^–O^2−^–Mn^2+^ super-exchange interaction [13,28]. This mechanism has three main influencing factors: the Fe^3+^–O^2−^–Mn^2+^ distance, the number of Fe^3+^–Mn^2+^ magnetic ion pairs and the number of oxygen ions as the Fe^3+^–O^2−^–Mn^2+^ interaction medium [30]. As approved by XPS, the concentration of Fe^3+^ ions increases with the Ho and Mn co-doping, while the concentration of oxygen vacancies is just the opposite. This predicates that the increasing in number of Fe^3+^–Mn^2+^ magnetic ion pairs and the number of oxygen ions in the Fe^3+^–O^2−^–Mn^2+^ interaction medium [13,39]. At the same time, the magnetization is highly sensitive to small changes in lattice constants [24]. The reduction of lattice constants (Figure 2c,d) will correspond to a shorter Fe^3+^–O^2−^–Mn^2+^ distance, which is also beneficial for improving the ferromagnetic coupling [24]. 

## 4. Conclusions

In summary, pristine, Ho-doped, Mn-doped and double-doped BFO thin films have been well fabricated on silicon substrates via a simple sol-gel process. The relationships among the structure, morphology as well as ion-modified magnetic properties of BFO thin film are systematically studies. XRD and Raman results preliminarily show that the Mn and Ho doping at Fe and Bi sites induces the structural transition in the parent BFO structure. The SEM and TEM images demonstrate that BHFMO thin film has a denser morphology with smaller particle sizes. The size of the particle is within the range of 25–45 nm. Therefore, it will lead to ferromagnetic behavior, resulting in incomplete uncompensated spins that are measurable. Magnetization measurements exhibited that the saturation magnetization (Ms) of BHFMO thin film (Ms = 58.62 emu/cm^3^) increases by approximately two times compared to BFO thin film (Ms = 31.37 emu/cm^3^) at room temperature. The enhancement of magnetic property might be associated to the destroying of the spatially modulated spin structures, the magnetically active characteristic of Ho^3+^ ions and generation of new exchange interactions. Consequently, adjusting the location of doped ions in BFO thin films can produce so much the better materials for diverse latent applications.

## Figures and Tables

**Table 1:** The content of metal elements in BiFeO_3_ (BFO), Bi_0.95_Ho_0.05_FeO_3_ (BHFO), BiFe_0.95_Mn_0.05_O_3_ (BFMO) and Bi_0.95_Ho_0.05_Fe_0.95_Mn_0.05_O_3_ (BHFMO) thin films.

Sample	Element	Concentration (mg/L)	Real Content (wt.%)	Theoretical Content (wt.%)
	Bi	0.133	66.5	66.8
**BFO**	Fe	0.035	17.5	17.9
	O	-	-	15.3
	Bi	0.127	63.5	63.9
**BHFO**	Fe	0.035	17.5	18.0
	Ho	0.005	2.5	2.7
	O	-	-	15.4
	Bi	0.132	66.0	66.8
**BFMO**	Fe	0.034	17.0	17.0
	Mn	0.001	0.5	0.9
	O	-	-	15.3
	Bi	0.127	63.5	63.9
	Ho	0.005	2.5	2.7
**BHFMO**	Fe	0.033	16.5	17.0
	Mn	0.001	0.5	0.9
	O	-	-	15.5

## References

[1] Hussain S., Hasanain S.K., Jaffari G.H., Ali N.Z., Siddique M., Shah S.I. (2017). Anomalous Temperature Dependence of Magnetic Coercivity and structure property correlations in Bi_0.75_A_0.25_FeO_3_ (A = Sr, Pb and Ba) system. J. Mater. Chem. C.

[2] Eerenstein W., Mathur N.D., Scott J.F. (2006). Multiferroic and magnetoelectric materials. Nature.

[3] Ramesh R. (2009). Materials science: Emerging routes to multiferroics. Nature.

[4] Si Y.H., Xia Y., Shang S.K., Xiong X.B., Zeng X.R., Zhou J., Li Y.Y. (2018). Enhanced Visible Light Driven Photocatalytic Behavior of BiFeO_3_/Reduced Graphene Oxide Composites. Nanomaterials.

[5] Shirolkar M.M., Li J.N., Dong X.L., Li M., Wang H.Q. (2017). Controlling the ferroelectric and resistive switching properties of BiFeO_3_ thin film prepared using sub-5 nm dimension nanoparticles. Phys. Chem. Chem. Phys..

[6] Quan Z.C., Hu H., Xu S., Liu W., Fang G.J., Li M.Y., Zhao X.Z. (2008). Surface chemical bonding states and ferroelectricity of Ce-doped BiFeO_3_ thin films prepared by sol-gel process. J. Sol-Gel Sci. Technol..

[7] Gu Y.H., Zhao J.G., Zhang W.Y., Zheng H.W., Liu L.M., Chen W.P. (2017). Structural transformation and multiferroic properties of Sm and Ti co-doped BiFeO_3_ ceramics with Fe vacancies. Ceram. Int..

[8] Chang L.Y., Tu C.S., Chen P.Y., Chen C.S., Schmidt V.H., Wei H.H., Huang D.J., Chan T.S. (2016). Raman vibrations and photovoltaic conversion in rare earth doped (Bi_0.93_-R_0.07_)FeO_3_ (R = Dy, Gd, Eu, Sm) ceramics. Ceram. Int..

[9] Jeon N., Rout D., Kim I.W., Kang S.J.L. (2011). Enhanced multiferroic properties of single-phase BiFeO_3_ bulk ceramics by Ho doping. Appl. Phys. Lett..

[10] Li Z.J., Hou Z.L., Song W.L., Liu X.D., Cao W.Q., Shao X.H., Cao M.S. (2016). Unusual continuous dual absorption peaks in Ca-doped BiFeO_3_ nanostructures for broadened microwave absorption. Nanoscale.

[11] Varshney D., Sharma P., Satapathy S., Gupta P.K. (2014). Structural, magnetic and dielectric properties of Pr-modified BiFeO_3_ multiferroic. J. Alloys Compd..

[12] Qi J., Zhang Y.L., Wang Y.H., Liu Y.Q., Wei M.B., Zhang J.K., Feng M., Yang J.H. (2017). Effect of Cr doping on the phase structure, surface appearance and magnetic property of BiFeO_3_ thin films prepared via sol-gel technology. J. Mater. Sci. Mater. Electron..

[13] Dong G.H., Tan G.Q., Luo Y.Y., Liu W.L., Ren H.J., Xia A. (2014). Structural transformation and multiferroic properties of single-phase Bi_0.89_Tb_0.11_Fe_1__−__x_Mn_x_O_3_ thin films. Appl. Surf. Sci..

[14] Rao T.D., Karthik T., Srinivas A., Asthana S. (2012). Study of structural, magnetic and electrical properties on Ho-substituted BiFeO_3_. Solid State Commun..

[15] Dutta D.P., Tyagi A.K. (2018). Effect of Sm^3+^ and Zr^4+^ codoping on the magnetic, ferroelectric and magnetodielectric properties of sonochemically synthesized BiFeO_3_ nanorods. Appl. Surf. Sci..

[16] Mukherjee A., Basu S., Green L.A.W., Thanh N.T.K., Pal M. (2015). Enhanced multiferroic properties of Y and Mn codoped multiferroic BiFeO_3_ nanoparticles. J. Mater. Sci..

[17] Lin F.T., Yu Q.Q., Deng L.Y., Zhang Z.J., He X.Y., Liu A.Y., Shi W.Z. (2017). Effect of La/Cr codoping on structural transformation, leakage, dielectric and magnetic properties of BiFeO_3_ ceramics. J. Mater. Sci..

[18] Chaturvedi S., Bag R., Sathe V., Kulkarni S., Singh S. (2016). Holmium induced enhanced functionality at room temperature and structural phase transition at high temperature in bismuth ferrite nanoparticles. J. Mater. Chem. C.

[19] Zheng Y.J., Tan G.Q., Xia A., Ren H.J. (2016). Structure and multiferroic properties of multi-doped Bi_1__−x_Er_x_Fe_0.96_Mn_0.02_Co_0.02_O_3_ thin films. J. Alloys Compd..

[20] Zhu Y.Y., Quan C.Y., Ma Y.H., Wang Q., Mao W.W., Wang X.F., Zhang J., Min Y.G., Yang J.P., Li X.A. (2017). Effect of Eu, Mn co-doping on structural, optical and magnetic properties of BiFeO_3_ nanoparticles. Mater. Sci. Semicond. Process..

[21] Islam M.R., Islam M.S., Zubair M.A., Usama H.M., Azam M.S., Sharif A. (2018). Evidence of superparamagnetism and improved electrical properties in Ba and Ta co-doped BiFeO_3_ ceramics. J. Alloys Compd..

[22] Kumar V., Singh S. (2016). Improved structure stability, optical and magnetic properties of Ca and Ti co-substituted BiFeO_3_ nanoparticles. Appl. Surf. Sci..

[23] Yuan X.Y., Shi L., Zhao J.Y., Zhou S.M., Li Y., Xie C.Z., Guo J.H. (2017). Sr and Pb co-doping effect on the crystal structure, dielectric and magnetic properties of BiFeO_3_ multiferroic compounds. J. Alloys Compd..

[24] Chen J.Y., Yao W., Yuan D. (2014). Combined effects of Bi deficiency and Mn substitution on the structural transformation and functionality of BiFeO_3_ films. J. Appl. Phys..

[25] Muneeswaran M., Lee S.H., Kim D.H., Jung B.S., Chang S.H., Jang J.W., Choi B.C., Jeong J.H., Giridharan N.V., Venkateswaran C. (2018). Structural, vibrational and enhanced magneto-electric coupling in Ho-substituted BiFeO_3_. J. Alloys Compd..

[26] Chen L.S., Zheng L.R., He Y.H., Zhang J., Mao Z.Q., Chen X. (2015). The local distortion and electronic behavior in Mn doped BiFeO_3_. J. Alloys Compd..

[27] Song G.L., Song Y.C., Su J., Song X.H., Zhang N., Wang T.X., Chang F.G. (2017). Crystal structure refinement, ferroelectric and ferromagnetic properties of Ho^3+^ modified BiFeO_3_ multiferroic. J. Alloys Compd..

[28] Chermahini M.D., Safaee I., Kazazi M., Shahraki M.M. (2018). Enhanced multiferroic properties of sono-synthesized BiFeO_3_ nanoceramics by co-doping of Sm and Mn elements. Ceram. Int..

[29] Raghavan C.M., Kim J.W., Kim S.S. (2014). Effects of (Dy, Zn) co-doping on structural and electrical properties of BiFeO_3_ thin films. Ceram. Int..

[30] Chai Z.J., Tan G.Q., Yue Z.W., Yang W., Guo M.Y., Ren H.J., Xia A., Xue M.T., Liu Y., Lv L. (2018). Ferroelectric properties of BiFeO_3_ thin films by Sr/Gd/Mn/Co multi-doping. J. Alloys Compd..

[31] Palkar V.R., John J., Pinto R. (2002). Observation of saturated polarization and dielectric anomaly in magnetoelectric BiFeO_3_ thin films. Appl. Phys. Lett..

[32] Cheng S.A., Zhang B.P., Zhao L., Wang K.K. (2018). Enhanced insulating and piezoelectric properties of 0.7BiFeO_3_ -0.3BaTiO_3_ lead-free ceramics by optimizing calcination temperature: Analysis of Bi^3+^ volatilization and phase structures. J. Mater. Chem. C.

[33] Lv J., Lou X.J., Wu J.G. (2016). Defect Dipoles-induced Poling Characteristics and Ferroelectricity of Quenched Bismuth Ferrite-based Ceramics. J. Mater. Chem. C.

[34] Dutta D.P., Jayakumar O.D., Tyagi A.K., Girija K.G., Pillai C.G.S., Sharma G. (2010). Effect of doping on the morphology and multiferroic properties of BiFeO_3_ Nanorods. Nanoscale.

[35] Shirolkar M.M., Hao C.S., Dong X.L., Guo T., Zhang L., Li M., Wang H.Q. (2014). Tunable multiferroic and bistable/complementary resistive switching properties of dilutely Li-doped BiFeO_3_ nanoparticles: An effect of aliovalent substitution. Nanoscale.

[36] Ahmmad B., Islam M.Z., Billah A., Basith M.A. (2016). Anomalous coercivity enhancement with temperature and tunable exchange bias in Gd and Ti co-doped BiFeO_3_ multiferroics. J. Phys. D Appl. Phys..

[37] Ederer C., Fennie C.J. (2008). Electric-field switchable magnetization via the Dzyaloshinskii-Moriya interaction: FeTiO_3_ versus BiFeO_3_. J. Phys. Condens. Matter..

[38] Liu Y.Q., Wang Y.J., Zhang J., Gao M., Zhang Y.J., Wei M.B., Yang J.H. (2015). Effect of Ho substitution on structure and magnetic property of BiFeO_3_ prepared by sol-gel method. Mater. Sci. Semicond. Process..

[39] Gotardo R.A.M., Silva E.F.R., Montanher D.Z., Santos G.M., Silva K.L., Cótica L.F., Santos I.A., Guo R., Bhalla A.S. (2017). Improved magnetic properties and structural characterizations in Mn doped 0.9BiFeO_3_-0.1BaTiO_3_ compositions. Scr. Mater..

